# Complete Genome Sequence of Prepandemic *Vibrio parahaemolyticus* BB22OP

**DOI:** 10.1128/genomeA.00002-12

**Published:** 2013-02-21

**Authors:** Roderick V. Jensen, Saylem M. DePasquale, Elizabeth A. Harbolick, Tian Hong, Alison L. Kernell, David H. Kruchko, Thero Modise, Cimarron E. Smith, Linda L. McCarter, Ann M. Stevens

**Affiliations:** a Department of Biological Sciences, Virginia Tech, Blacksburg, Virginia, USA; b Department of Microbiology, University of Iowa, Iowa City, Iowa, USA

## Abstract

The number of inflammatory gastroenteritis outbreaks due to the food-borne pathogen *Vibrio parahaemolyticus* is rising sharply worldwide and in the United States in particular. Here we report the complete, annotated genome sequence of the prepandemic *V. parahaemolyticus* strain BB22OP and make some initial comparisons to the complete genome sequence for pandemic strain RIMD2210633.

## GENOME ANNOUNCEMENT

*Vibrio parahaemolyticus* is found in marine-associated aquatic environments freely living, attached to abiotic and biotic (e.g., plankton and shellfish) surfaces, or associated with marine animal hosts (1, 2). It is also a major cause of seafood-borne gastroenteritis in humans, occasionally causing death (2–4). Since the complete genome sequence of clinical isolate RIMD2210633 was published (5), draft genome sequences for several other *V. parahaemolyticus* strains have been announced (6–8) or are available online (http://www.genomesonline.org). Here we announce the complete genome sequence of *V. parahaemolyticus* BB22OP, a Bangladesh environmental isolate from the early 1980s (9). This strain, with an O4:K8 serotype, has been extensively studied with respect to swimming and swarming motility, biofilm development, and phase variation (10–15).

A single opaque colony of the *V. parahaemolyticus* BB22OP strain LM5312 (16) was grown in heart infusion (HI) broth (13) at 30°C overnight. The Qiagen DNeasy blood and tissue kit protocol for Gram-negative bacteria was utilized to extract DNA, which was sequenced using a Roche/454 GS FLX titanium system. A 200× coverage of the genome was achieved and *de novo* sequence assembly of the reads was then performed using Roche/454 Newbler software. MIRA (http://www.chevreux.org/projects_mira.html) *de novo* assembly was performed to assist in filling in gaps in the genome assembly. Alignment to the RIMD2210633 reference genome sequence using the Lasergene 8 software package also facilitated complete genome assembly into circular chromosomes 1 and 2 of lengths 3.297 Mbp and 1.806 Mbp, respectively. When necessary, primers were designed and traditional Sanger sequencing was performed to confirm putative assembly junctions. The BB22OP genome contains eleven ∼5-kbp rRNA/tRNA clusters at the same locations as those in RIMD2210633. Since unique Roche/454 or Sanger sequences could not span these regions, the corresponding RIMD2210633 sequences were used to help fill these gaps.

The genome sequence was annotated on the Rapid Annotation using Subsystem Technology (RAST) server (http://rast.nmpdr.org/) with the help of the GenBank annotation of RIMD2210633. Annotation of sequences unique to BB22OP was performed using both GeneMark-P^∗^ and Genemark.hmm-P (http://exon.biology.gatech.edu). Coding sequences for 2,973 genes on chromosome 1 and 1,653 genes on chromosome 2 were identified. Overall, there is extensive homology between the two strains; >90% of the coding sequences have the same annotation, length, and relative position. There are ∼300 genes novel to BB22OP, and ∼400 genes novel to RIMD2210633. Many of the novel genes appear to be remnants of transposons or phages. With respect to potential virulence traits, and like RIMD2210633, the BB22OP genome encodes thermostable direct hemolysin (Tdh) and two type-3 and two type-6 secretion systems, and it lacks genes encoding thermostable direct hemolysin-related hemolysin (Trh) and urease (Ure) (17). BB22OP also lacks prophage f237 (18) and genomic islands VPaI-1 and VPaI-3 to VPaI-6 (19). The two strains differ in the superintegron on chromosome 1 (∼90 genes) and in two small blocks encoding the O and K antigens (∼12 genes each). With BB22OP being a prepandemic isolate, the genome sequence will provide an important anchor for future comparative genomic and virulence studies.

### Nucleotide sequence accession numbers. 

The complete, annotated genome sequence for *V. parahaemolyticus* BB22OP strain LM5312 is available in GenBank under accession numbers CP003972 (BB22OPChr1) and CP003973 (BB22OPChr2).
